# P-12. Impact of Development and Implementation of System Wide Guidelines on Management of Uncomplicated Gram-Negative Bacteremia at a Community Hospital

**DOI:** 10.1093/ofid/ofaf695.243

**Published:** 2026-01-11

**Authors:** Darshan Patel, Lucy S Witt, Hasan Shabbir

**Affiliations:** Emory Johns Creek Hospital, Acworth, GA; Emory University, Atlanta, Georgia; Emory Universtiy, Atlanta, Georgia

## Abstract

**Background:**

Gram-negative bacteremia (GNB) is associated with considerable morbidity and mortality. Recent data suggests that shorter courses and early step-down to oral (PO)antibiotics have equivalent outcomes to intravenous (IV) therapy in uncomplicated cases. Oral antibiotics can reduce hospital length of stay (LOS) as well as complications from parenteral therapy. We evaluated clinical outcomes of patients with uncomplicated GNB pre and post implementation of systemwide treatment guideline in May 2024 at our medium sized community-academic partnership hospital.

Figure 1.

**Figure:**
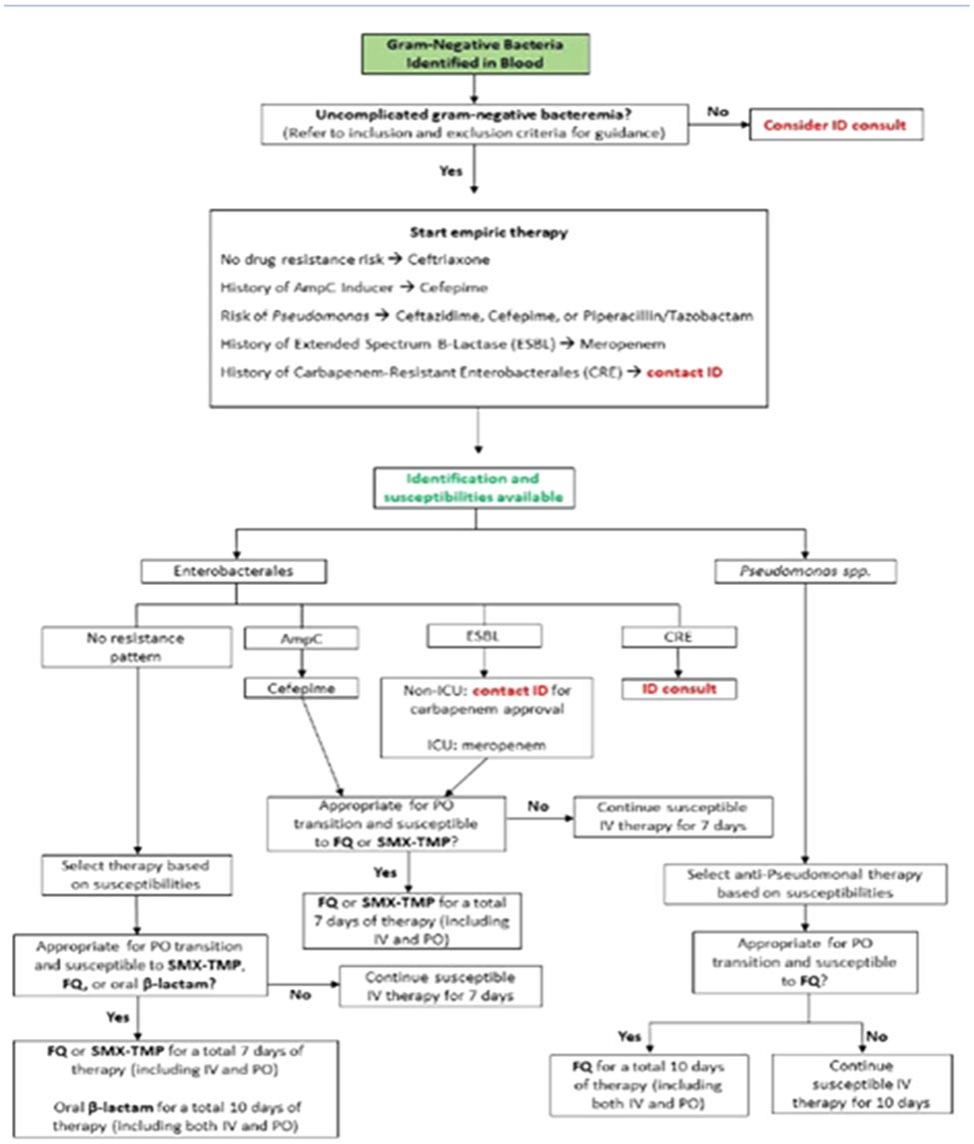

**Table 1.**
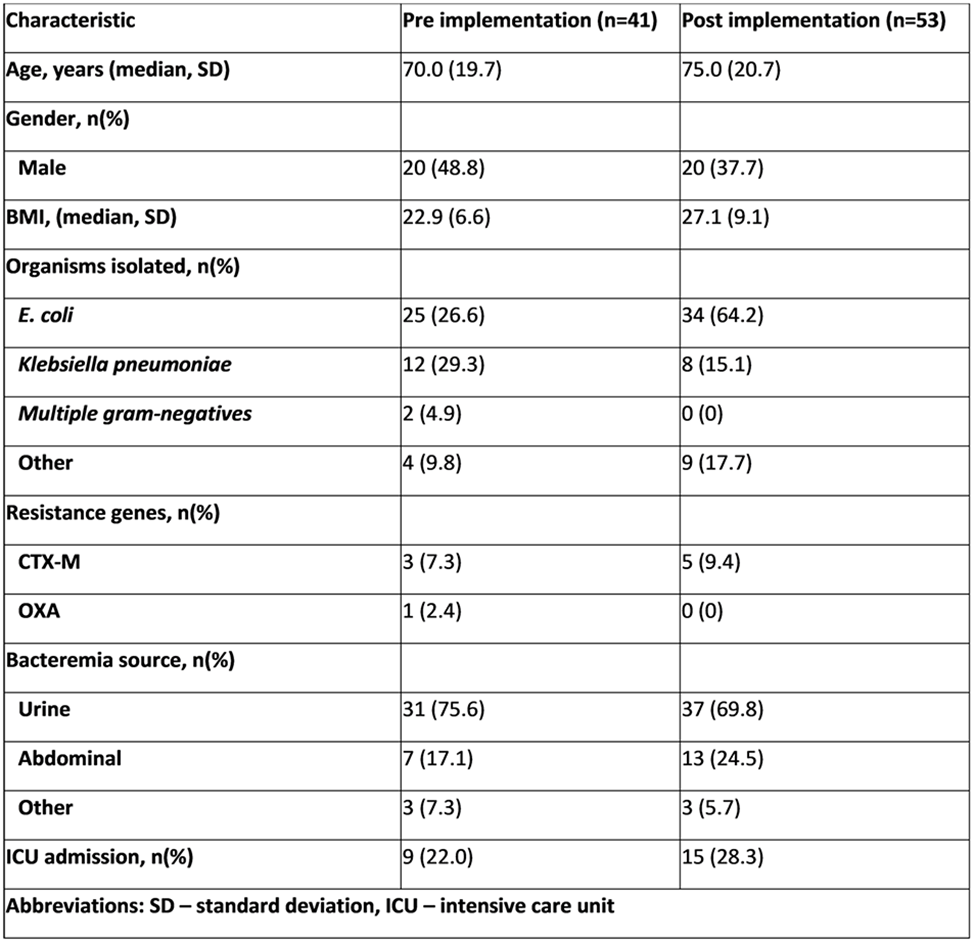
Demographics and treatment characteristics before and after implementation of uncomplicated Gram-negative bacteremia guidelines

**Methods:**

Basic demographic and clinical data was collected on patients with uncomplicated GNB in the 4 months before and 7 months after guideline implementation. The primary outcome was total duration of antibiotic therapy. Secondary outcomes included number of patients who received < 10 days of therapy, time to PO stepdown, duration of IV antibiotics, LOS, and 30-day hospital readmission.

Table 2.

**Figure:**
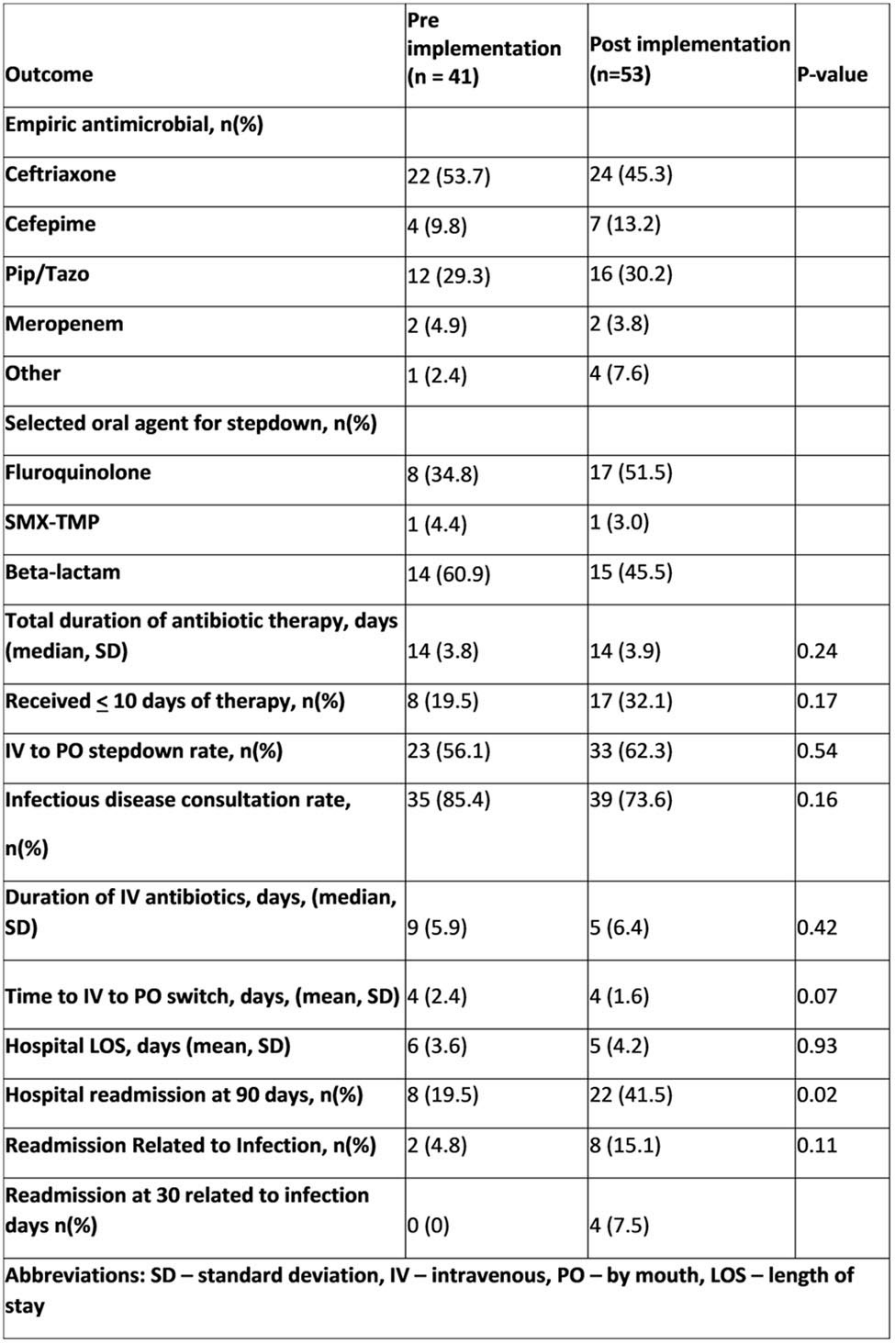

Treatment Outcomes by period before and after Gram-negative guideline implementation

**Results:**

94 patients (41 pre- ,53 post-implementation) met criteria for uncomplicated GNB per internal guidelines. Ceftriaxone was the most commonly use empiric antibiotic in both periods. *E.coli* and *Klebsiella pneumoniae* were the most frequently isolated pathogens. Urinary and abdominal sources were the most common sources of bacteremia. No difference was observed in median duration of therapy after guideline implementation, however more patients in the post- introduction period were treated for < 10 days (8 v 17; p=0.17). In addition, more patients were transitioned to oral antibiotics (62% vs. 56%; p=0.54). Median duration of IV antibiotic therapy (5 vs. 9 days; p=0.42) and LOS (5 vs. 6 days; p=0.93) were also shorter post guideline introduction. An increase in hospital readmission at 90 days was observed, but a majority of these were not infection related.

**Conclusion:**

After introducing a guideline for treatment of uncomplicated GNB, patients in our hospital received shorter duration of IV therapy and more frequently received PO antibiotics. Our study was underpowered to assess whether these observations were statistically significant. Future investigation will expand the period evaluated both before and after guideline introduction to observe persistence of positive trends and increase statistical power.

**Disclosures:**

Lucy S. Witt, MD, MPH, Merck & Co: Grant/Research Support

